# Perceived GenAI competency and university teachers’ intention to continue using generative artificial intelligence: Extending the technology acceptance model

**DOI:** 10.1371/journal.pone.0358596

**Published:** 2026-09-17

**Authors:** Qiusu Wang

**Affiliations:** School of Education, Beijing Institute of Technology, Beijing, China; Shanghai International Studies University, CHINA

## Abstract

Generative artificial intelligence (GenAI) is reshaping higher education, and understanding teachers’ intention to continue using GenAI is increasingly important in this context. Grounded in the technology acceptance model (TAM), this study examines how different dimensions of teachers’ perceived GenAI competency are associated with perceived ease of use (PEOU), perceived usefulness (PU), and GenAI use continuance intention (GenAI-UCI). Using survey data from 615 university teachers in Qingdao, China, structural equation modeling was employed to examine the hypothesized relationships among perceived GenAI competency dimensions, TAM-based beliefs, and continuance intention. Results showed that perceived GenAI competency dimensions related to teaching, research, and professional engagement were positively associated with PU, whereas perceived basic understanding showed a small and non-robust association with PEOU. PU was positively associated with GenAI-UCI, whereas PEOU showed no significant direct association with GenAI-UCI. Overall, the findings suggest that perceived GenAI competency is not a homogeneous capability; rather, different competency dimensions were associated with different TAM-based beliefs within the proposed model. The findings highlight the importance of teachers’ perceptions of GenAI usefulness in their professional practices for understanding continuance intention in higher education.

## Introduction

### Generative artificial intelligence in higher education

Higher education is undergoing rapid transformation as a result of generative artificial intelligence (GenAI), which is changing how academic knowledge is produced, taught, and learned [1,2]. Through the automated generation of text, instructional materials, assessments, and feedback, GenAI tools are increasingly embedded in teaching practices [3,4]. These technologies may offer potential to improve educational efficiency, reduce repetitive workloads, and support personalized and scalable learning environments [5]. GenAI has also been discussed as having potential to support access to learning, adaptive content delivery, and pedagogical practices [6,7]. Emerging evidence further shows that GenAI can support teachers in lesson planning, instructional design, and task development, with potential implications for interdisciplinary learning, student engagement, and more adaptive and data-informed educational practices [8,9].

Despite these opportunities, the responsible and ethical integration of GenAI in higher education remains challenging. Concerns regarding academic integrity, ethical use, algorithmic bias, and over-reliance on automated outputs may threaten educational quality if left unaddressed [10,11]. Moreover, the rapid adoption of GenAI requires educators to continuously develop their perceived competencies for responsible and pedagogically sound use [12]. Consequently, teachers’ continuance intention toward GenAI may be related not only to technological considerations but also to their perceived capabilities and willingness to continue using these tools [13,14]. Understanding the factors associated with teachers’ continuance intention toward GenAI, rather than initial adoption alone, is therefore important for examining continued GenAI use in higher education.

### Teachers’ generative artificial intelligence acceptance and perceived competency in higher education

To help understand teachers’ use of emerging technologies, such as GenAI, the technology acceptance model (TAM) provides a well-established theoretical foundation. The TAM posits that perceived usefulness (PU) and perceived ease of use (PEOU) are key beliefs associated with individuals’ behavioral intention to use technology [15]. In educational contexts, the TAM has been widely applied to examine teachers’ acceptance of digital tools, online learning platforms, and AI applications across diverse settings [16,17]. Within the context of GenAI, PU refers to the extent to which teachers believe that these tools can support teaching effectiveness, instructional quality, and productivity, whereas PEOU refers to the degree to which GenAI systems are perceived as accessible, user-friendly, and easy to integrate into pedagogical practices [15].

Applying TAM to teachers’ use of GenAI provides insight into how individual perceptions and competencies are associated with technology acceptance beliefs and continuance intention in higher education. Teachers’ acceptance of GenAI has been discussed in relation to the potential integration of these technologies into educational practices [18]. Teachers’ perceptions of GenAI may also be relevant to their intention to continue using these tools in instructional practices [19,20]. However, the present study does not examine educational sustainability outcomes such as equity, instructional quality, or institutional transformation [21]. Therefore, rather than framing GenAI acceptance in terms of sustainable education systems, this study focuses specifically on the associations between teachers’ perceived GenAI competency, TAM-based beliefs, and GenAI use continuance intention. Examining factors associated with teachers’ PU and PEOU of GenAI is therefore important for understanding continuance intention in higher education. However, the TAM primarily focuses on cognitive beliefs about technology and provides limited insight into how teachers’ perceived capabilities are associated with these beliefs [22].

Teachers’ perceived GenAI competency refers to their self-assessed knowledge, skills, and confidence in integrating GenAI into educational practice in a pedagogically sound and ethically responsible manner [23]. As GenAI becomes increasingly embedded in higher education, teachers are expected not only to understand its technological aspects but also to consider its operational functions, pedagogical applications, and ethical implications in educational contexts [24]. In this sense, perceived GenAI competency extends beyond basic digital literacy and represents a multidimensional perceived capability that integrates technological, pedagogical, and ethical dimensions [23–26]. Several established frameworks provide relevant guidance for conceptualizing such competencies. The United Nations Educational, Scientific and Cultural Organization AI competency framework for teachers [25] and the European Framework for the Digital Competence of Educators [26] both emphasize technological, pedagogical, and ethical dimensions, with particular attention to responsible AI use and human oversight in educational practice.

Teachers’ perceived GenAI competency is increasingly recognized as a factor associated with the use of AI in higher education. Previous research has shown that teachers with higher levels of perceived digital and AI-related competence are more likely to engage in innovative instructional practices and adapt to emerging educational technologies [27]. Higher perceived competency may also be associated with teachers’ ability to critically evaluate AI-generated outputs, select appropriate instructional applications, and consider academic integrity in teaching and assessment practices [28]. Perceived competency has also been discussed in relation to instructional design, confidence in technology use, and the pedagogical value perceived by teachers [29]. In contrast, lower levels of perceived competency may be associated with more limited engagement with GenAI tools and lower perceived pedagogical value. These findings provide a basis for examining perceived GenAI competency as a multidimensional construct associated with teachers’ TAM-based beliefs and GenAI use continuance intention in higher education.

### Research gaps and objectives

Despite growing interest in GenAI use in higher education, how teachers’ perceived competencies are associated with technology acceptance beliefs and continuance intention remains insufficiently understood. In particular, it remains unclear whether different dimensions of perceived GenAI competency are associated with PU and PEOU within the TAM framework. First, previous research has mainly examined teachers’ acceptance and adoption of GenAI, whereas less attention has been given to continuance intention within a competency-informed framework [30]. As discussed above, the TAM emphasizes PU and PEOU as key beliefs associated with technology use intention. However, this perspective provides limited insight into how teachers’ perceived competencies are related to these cognitive beliefs. Second, although perceived GenAI competency has been increasingly recognized as relevant to effective AI integration in education, its relationship with teachers’ technology acceptance remains underexplored [31]. Perceived GenAI competency reflects teachers’ self-perceived technological, pedagogical, and ethical capabilities [31], providing a theoretical basis for examining whether different competency dimensions are associated with perceptions of the usefulness and ease of use of GenAI tools.

To address these gaps, this study investigates how teachers’ perceived GenAI competency is associated with their intention to continue using GenAI in higher education. By integrating perceived GenAI competency into TAM, the study proposes a framework linking perceived competency dimensions, technology acceptance beliefs, and GenAI use continuance intention, thereby extending the TAM in the context of emerging AI in education. This framework examines whether different dimensions of perceived GenAI competency are related to teachers’ cognitive evaluations of GenAI and their intention to continue using it in teaching practice. The study contributes to the literature by examining perceived GenAI competency as a multidimensional construct associated with technology acceptance beliefs and by providing insights into the indirect pathways linking perceived competencies with GenAI continuance intention within the specified model. These findings may inform professional development and the responsible integration of GenAI in higher education. Beyond the current empirical context, the proposed framework provides a basis for future research to examine whether similar patterns of association emerge across diverse educational levels, cultural contexts, and emerging AI applications.

## Theoretical framework and hypothesis development

### Technology acceptance model

The TAM is a foundational model for understanding technology acceptance, emphasizing PU and PEOU as core determinants of users’ behavioral intention to use technology [15]. The model posits that individuals’ behavioral intention to use a technology is associated with PU and PEOU [15]. As AI in education continues to evolve rapidly, the TAM has emerged as one of the most frequently used models for examining teachers’ acceptance of AI technologies in educational contexts [32]. In educational research, numerous studies have applied the TAM across various settings, including online learning environments, learning management systems, and AI-supported teaching tools, demonstrating its relevance to understanding teachers’ technology acceptance [33–35].

In the context of this study, the TAM provides a useful theoretical lens for understanding teachers’ GenAI use continuance intention in higher education, particularly when combined with individual factors such as teachers’ perceived competencies. This perspective highlights the continued relevance of the TAM while providing a basis for examining how perceived GenAI competency is associated with TAM-based beliefs and GenAI use continuance intention in an emerging AI context.

### Generative artificial intelligence basic understanding and perceived ease of use

Perceived GenAI basic understanding (GenAI-BU) refers to teachers’ self-assessed knowledge of GenAI, including their perceived understanding of fundamental concepts, functional applications, and ethical considerations related to responsible use in educational contexts [31]. Prior research has shown that users’ technological knowledge and familiarity with digital tools are positively associated with PEOU, as greater understanding may be related to smoother interaction with technology and lower perceived complexity in system use [32,33]. In the context of AI, ethical awareness may also be associated with greater confidence in using AI technologies, particularly in relation to uncertainty surrounding data privacy, algorithmic bias, and responsible use [34,35]. Therefore, teachers with higher perceived GenAI-BU may report higher PEOU of GenAI tools. Accordingly, the following hypothesis is proposed:

H1. Teachers’ perceived GenAI-BU is positively associated with PEOU.

### Generative artificial intelligence teaching and perceived usefulness

Perceived GenAI teaching (GenAI-TE) competency refers to teachers’ self-assessed capability to integrate GenAI into instructional practices, including student interaction, learning design, and assessment activities [31]. This competency reflects teachers’ confidence in using GenAI for lesson planning, instructional material development, personalized learning, and formative assessment [36,37]. Prior research has shown that teachers’ pedagogical use of digital technologies is positively associated with PU, as technologies may be perceived as more valuable when they are relevant to instructional efficiency and learning outcomes [38,39]. In the context of GenAI, teachers who perceive themselves as capable of applying AI in instructional processes may be more likely to perceive benefits for teaching and student learning [17]. Accordingly, higher perceived GenAI teaching competency is expected to be positively associated with perceptions of GenAI usefulness. Therefore, the following hypothesis is proposed:

H2. Teachers’ perceived GenAI-TE competency is positively associated with PU.

### Generative artificial intelligence research and perceived usefulness

Perceived GenAI research (GenAI-RE) competency refers to teachers’ self-assessed capability to use GenAI to support research design and data analysis processes, including formulating research topics, selecting appropriate methods, and analyzing and interpreting research data [31]. This competency reflects teachers’ confidence in applying GenAI tools to support research planning, analytical processes, and research-related tasks [40]. Prior research has shown that the use of digital technologies in academic work is positively associated with PU, as such tools may be perceived as valuable for supporting research efficiency and the quality of academic outputs [41]. In the context of GenAI, teachers who perceive themselves as capable of applying AI tools in research processes may be more likely to recognize their potential value for supporting complex analytical tasks and research productivity [42]. Accordingly, higher perceived GenAI research competency may be associated with stronger perceptions of GenAI usefulness. Therefore, the following hypothesis is proposed:

H3. Teachers’ perceived GenAI-RE competency is positively associated with PU.

### Generative artificial intelligence professional engagement and perceived usefulness

Perceived GenAI professional engagement (GenAI-PE) refers to teachers’ perceived capability and preparedness to engage in ongoing GenAI-related professional learning and knowledge updating [31]. It reflects teachers’ perceived ability to identify and address knowledge and skill gaps, seek relevant learning opportunities, and remain informed about emerging developments in GenAI [43]. Prior research has shown that engagement with digital technologies in professional contexts is positively associated with PU [44], as such technologies may be perceived as valuable for accessing up-to-date knowledge, supporting continuous learning, and developing professional competence [45]. In the context of GenAI, teachers who perceive themselves as capable of engaging in ongoing AI-related professional learning may be more likely to recognize its potential value for knowledge development and professional growth [46]. Accordingly, higher perceived GenAI-PE may be associated with stronger perceptions of GenAI usefulness. Therefore, the following hypothesis is proposed:

H4. Teachers’ perceived GenAI-PE is positively associated with PU.

### Perceived ease of use and perceived usefulness

Perceived ease of use refers to the degree to which individuals believe that using a particular technology would be free of effort, whereas PU reflects the extent to which they believe that using the technology would enhance their performance [15]. Within TAM, PEOU is considered to be positively associated with PU, as technologies perceived as easier to use may also be perceived as more useful due to lower perceived cognitive effort and greater perceived efficiency in task performance. When users perceive a technology as easy to operate, they may be more likely to recognize its functional benefits and evaluate its usefulness positively [47]. In the context of GenAI in education, teachers who perceive GenAI tools as easy to use may be more likely to recognize their potential value for teaching, research, and professional activities. Therefore, PEOU is expected to be positively associated with PU, leading to the following hypothesis:

H5. PEOU is positively associated with PU.

### Perceived usefulness and generative artificial intelligence-use continuance intention

Within TAM, PU is an important predictor of users’ behavioral intention, as it reflects the extent to which individuals believe that a technology can enhance their performance [15]. When users perceive a technology as valuable for supporting task efficiency and desired outcomes, they may report stronger intentions to continue using it [48]. In the context of GenAI in education, teachers who perceive GenAI tools as useful for teaching, research, and professional activities may report stronger intentions to continue using them in future practice. Therefore, PU is expected to be positively associated with teachers’ GenAI-use continuance intention (GenAI-UCI), leading to the following hypothesis:

H6. Perceived usefulness is positively associated with GenAI-UCI.

### Perceived ease of use and generative artificial intelligence-use continuance intention

Within TAM, PEOU is considered an important predictor of behavioral intention [15]. In the context of continuance intention, technologies perceived as easier to use may be associated with lower cognitive and operational effort and stronger intentions to continue using them. When users perceive a technology as easy to operate, they may report less frustration and greater confidence in its use, which may be related to stronger intentions to continue using it [49]. In the context of GenAI in education, teachers who perceive GenAI tools as easy to use may report stronger intentions to continue using them in teaching, research, and professional activities. Therefore, PEOU is expected to be positively associated with teachers’ GenAI-UCI, leading to the following hypothesis:

H7. PEOU is positively associated with GenAI-UCI.

**Fig 1:**
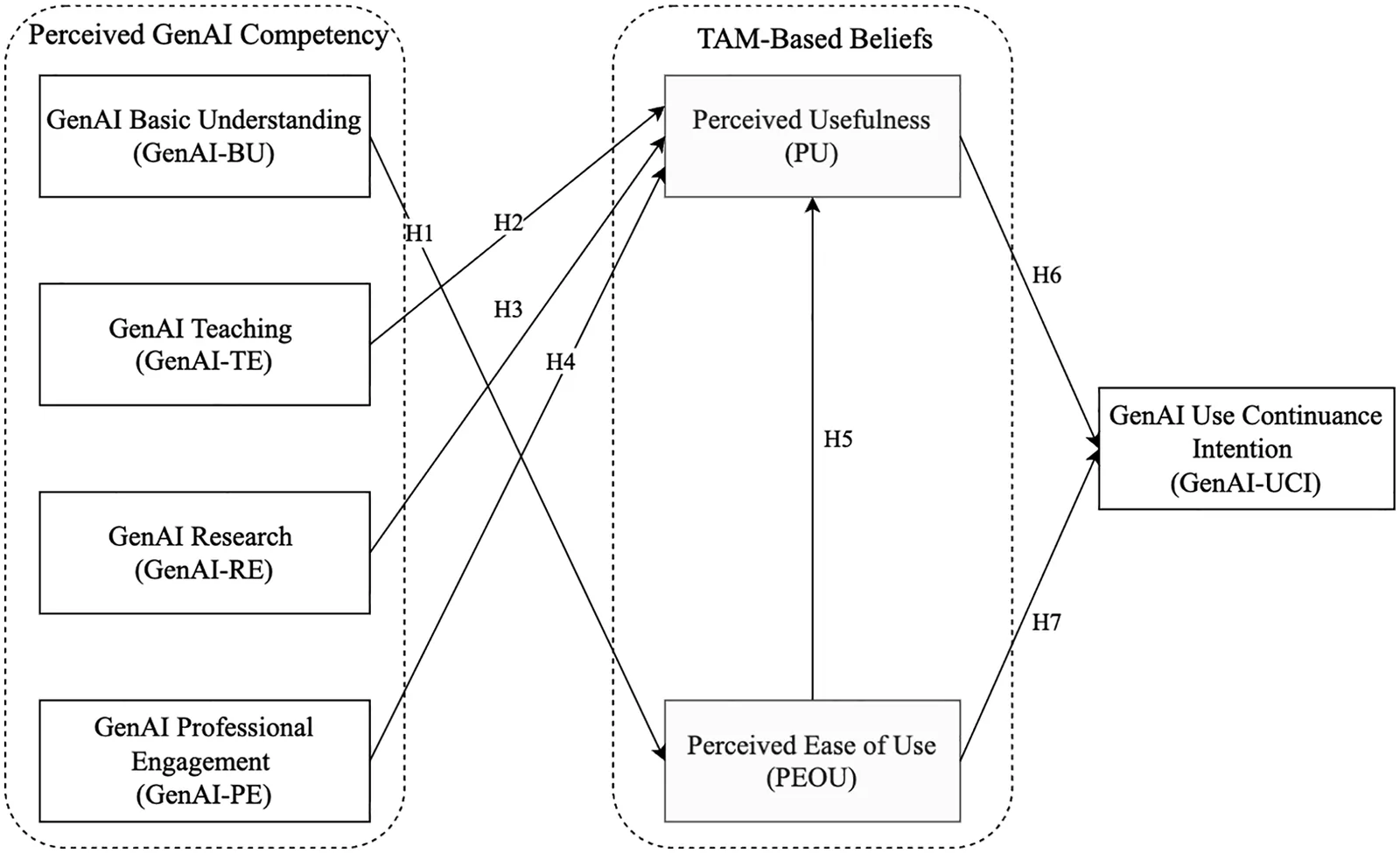
Proposed research model and hypotheses.

The conceptual framework and hypothesized relationships among the study variables are presented in Fig 1.

## Materials and methods

### Research instruments

The survey used in this study comprised two sections. The initial section focuses on collecting demographic data to obtain basic information about participants, such as gender, teaching experience, and frequency of GenAI use. The second part comprises multiple sub-scales designed to measure seven specific factors: GenAI-BU, GenAI-TE, GenAI-RE, GenAI-PE, PU, PEOU, and GenAI-UCI. The questionnaire uses a 5-point Likert scale, with 1 indicating “strongly disagree” and 5 indicating “strongly agree.”

The GenAI-BU, GenAI-TE, GenAI-RE, and GenAI-PE sub-scales collectively represent teachers’ perceived GenAI competency. The measurement items were adapted from the GenAI Competency Self-Efficacy Scale for Teachers and Researchers (GAICS-TR) created by Xia et al. [31]. The four dimensions were retained as perceived GenAI competency because the instrument was originally developed and validated as a multidimensional self-report measure of teachers’ perceived competence rather than objectively assessed competence. The GenAI-BU sub-scale (comprising 7 items) measures teachers’ self-perceived understanding of GenAI, including their knowledge of GenAI concepts and applications, as well as their awareness of ethical and responsible use in educational contexts. The GenAI-TE sub-scale (comprising 12 items) assesses teachers’ self-perceived capability in supporting student interaction, designing learning resources, and facilitating assessment in GenAI-enhanced educational environments. The GenAI-RE sub-scale (comprising 8 items) captures teachers’ perceived capability to support research design and conduct data analysis in GenAI-enhanced academic contexts. The GenAI-PE sub-scale (comprising 8 items) captures teachers’ perceived capability and preparedness to engage in GenAI-related professional learning and knowledge updating. It reflects their perceived ability to identify and address knowledge and skill gaps, seek relevant learning opportunities, and remain informed about emerging developments in GenAI. The term “professional engagement” in this construct refers to perceived capability and preparedness for professional learning rather than actual participation in professional development activities. Although some items refer to professional-learning activities, these activities are conceptualized as indicators of perceived capability and preparedness for professional development, rather than as measures of actual participation frequency or behavioral engagement. Consistent with the original conceptualization of the GAICS-TR [31], GenAI-PE was therefore retained as a dimension of perceived GenAI competency.

Regarding the TAM framework, the PU and PEOU scales (each comprising 6 items) were adapted from Davis [15]. The wording of the original TAM items was modified by replacing the general technology context with GenAI in higher education while preserving the original construct meanings. The GenAI-UCI sub-scale (comprising 8 items) was derived from the measurement tools developed by Chai et al. [50] and Bhattacherjee [51]. The PU and PEOU scales measure teachers’ beliefs about the potential usefulness of GenAI and the extent to which GenAI is perceived as easy to use, respectively. The GenAI-UCI scale assesses teachers’ future-oriented intention to continue using GenAI in professional practice. The items focus on continued involvement with GenAI following prior exposure or use, including maintaining awareness of GenAI developments and further developing GenAI-related skills. Although some items involve professional learning or skill development, these activities are treated as future-oriented manifestations of continuance intention rather than as measures of professional-development engagement itself. Thus, GenAI-UCI reflects intention to continue or extend GenAI use, whereas GenAI-PE captures perceived capability and preparedness for ongoing professional learning within the perceived competency framework. Table 1 presents the measurement constructs, number of items, sample items, and corresponding sources used in this study.

**Table 1:** Constructs, items, and measurement sources.

Construct	Number of Items	Sample Item	References
**GenAI Basic Understanding**	7	I can identify which tools belong to GenAI. (GenAI-BU1)	Xia et al. [31].
**GenAI in Teaching**	12	I can use GenAI to enhance the interactivity and engagement of classroom discussions. (GenAI-TE1)	Xia et al. [31].
**GenAI in Research**	8	I can use GenAI to help me formulate research topics. (GenAI-RE1)	Xia et al. [31].
**GenAI Professional Engagement**	8	I can use GenAI tools to identify and address knowledge and skill gaps in my teaching. (GenAI-PE1)	Xia et al. [31].
**Perceived Usefulness**	6	Using generative AI in my work would enable me to accomplish tasks more quickly. (PU1)	Davis [15]
**Perceived Ease of Use**	6	Learning to operate generative AI would be easy for me. (PEOU1)	Davis [15]
**GenAI Use Continuance Intention**	8	I intend to check updates about generative AI frequently. (GenAI-UCI1)	Chai et al. [50]; Bhattacherjee [51]

The questionnaire was administered in Chinese. All English-language scales were translated into Chinese using a forward–backward translation procedure. Two bilingual experts independently translated the original items into Chinese, after which an independent bilingual expert back-translated them into English. Any discrepancies were discussed until conceptual equivalence was achieved.

### Participants and ethical considerations

Participants were recruited from multiple higher education institutions in Qingdao, China, representing diverse institutional contexts. They comprised university teachers engaged in teaching activities at the participating institutions. In line with national policy priorities, Chinese higher education institutions are increasingly integrating AI into teaching, research, and campus management, particularly following the introduction of the “AI + Education” action plan [52]. Qingdao was selected as the research site because it provides a diverse higher education context in which GenAI is increasingly relevant to teaching and research practices [53].

A convenience sampling method was utilized to recruit participants. Participants were required to have prior familiarity with AI tools and teaching experience so that they could meaningfully respond to the GenAI-related items. Data were collected from three public comprehensive universities and three private comprehensive universities in Qingdao, China, through an online questionnaire distributed via the Chinese survey platform Wenjuanxing. The questionnaire was administered in Chinese, and participants completed the survey online at their own convenience. The questionnaire required approximately 10–15 minutes to complete. Data collection was conducted between January 2026 and February 2026 during the regular academic semester. The survey link was distributed through university administrative mailing lists and faculty WeChat groups. The six participating universities contributed 74, 98, 121, 82, 153, and 87 valid responses, respectively, yielding a total of 615 valid participants.

A total of 708 responses were initially received. Following data screening, 93 responses were excluded. Specifically, 41 responses were excluded because the questionnaire was completed in less than 5 minutes, 49 were excluded because respondents selected the same response option across all Likert-scale items (straight-lining), and 3 were excluded because the response records were completely identical to other records in the dataset. The < 5-minute exclusion criterion was established based on the estimated completion time from 30 pilot surveys. The survey was configured to allow each response identifier to be used only once. In addition, response records were compared after data collection, and records with completely identical responses across the questionnaire were excluded. The final sample comprised 615 valid responses, corresponding to a valid response rate of 86.9%.

**Fig 2:**
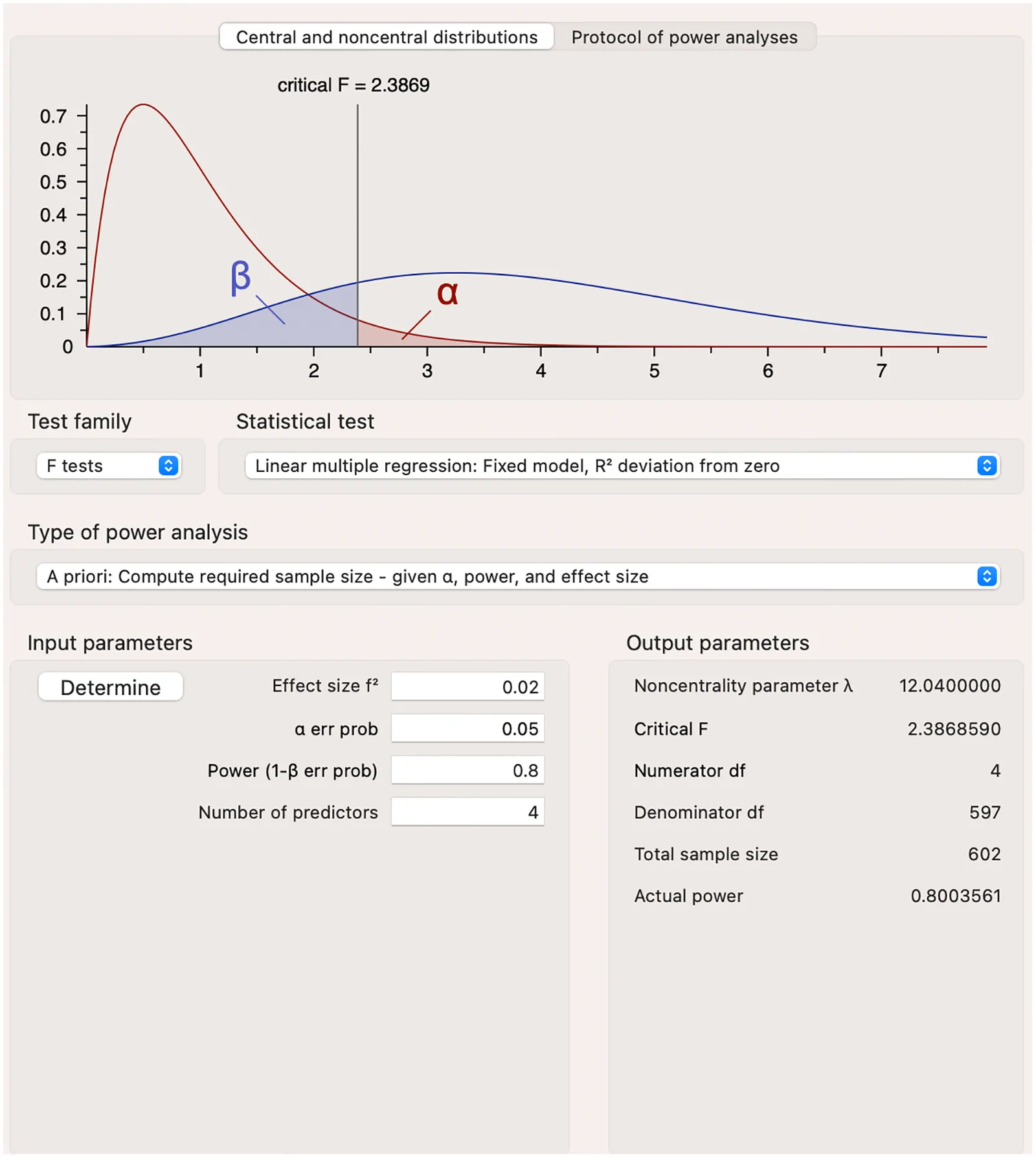
A priori power analysis and sample size calculation using G*Power.

An a priori power analysis was conducted using G*Power 3.1 with the test family “F tests” and the statistical test “Linear multiple regression: Fixed model, R² deviation from zero.” A small effect size (f² = 0.02), α = 0.05, and a statistical power of 0.80 were specified (Fig 2). Four predictors were specified, corresponding to the maximum number of predictors of an endogenous construct in the proposed structural model (i.e., GenAI-TE, GenAI-RE, GenAI-PE, and PEOU predicting PU). The analysis indicated a minimum required sample size of 602. The final valid sample of 615 participants exceeded the a priori minimum requirement of 602. The small effect-size assumption (f² = 0.02) was selected because the proposed model extends the TAM by incorporating teachers’ perceived GenAI competency as an individual-level antecedent, for which empirical evidence regarding its associations with TAM beliefs remains limited. Given the exploratory nature of these newly specified relationships and the expectation that individual competency factors would account for a relatively modest proportion of variance in technology acceptance beliefs, a conservative small-effect assumption was adopted.

Table 2 presents the demographic characteristics of the participating university teachers. The sample comprised 62.1% female and 37.9% male participants. The largest proportion of respondents had 6–10 years of teaching experience (42.4%). Regarding GenAI usage frequency, 59.2% of participants reported using GenAI daily, while 33.8% reported using it 1–2 times per week.

**Table 2:** Demographic characteristics of the participants (*n* = 615).

Demographic Characteristics		Number of Participants (*n*)	Percentage (%)
**Gender**	Male	233	37.9
	Female	382	62.1
**Teaching Experience**	1–5 years	173	28.1
	6–10 years	261	42.4
	11–15 years	140	22.8
	16–20 years	37	6.0
	More than 20 years	4	0.7
**GenAI Usage Frequency**	Rarely (less than once a month)	7	1.1
	Occasionally (1–3 times per month)	36	5.9
	Often (1–2 times per week)	208	33.8
	Very often (daily)	364	59.2

Ethical approval for this study was obtained from the Institutional Review Board of Beijing Institute of Technology (Approval No. 2025−12), with all procedures conducted in accordance with the Declaration of Helsinki. All participation was voluntary, and respondents were first presented with a detailed explanation of the study, including its objectives, their right to withdraw at any stage without penalty, and assurances regarding confidentiality and anonymity. The study did not collect any personally identifiable information, and all responses were stored securely and used exclusively for academic research purposes. Consent was obtained within the online survey platform. Specifically, participants were required to read the consent information and proceed by clicking the “Next” button, which functioned as an indication of implied consent for participation, data analysis, and the dissemination of anonymized results.

### Data analysis and reliability

Data analysis was conducted using IBM SPSS Statistics 29 and AMOS 26. Descriptive statistics were first calculated to describe participant characteristics and examine the distribution of all variables, including means, standard deviations, skewness, and kurtosis. Common method bias was assessed using Harman’s single-factor test [54] and the unmeasured latent method construct (ULMC) approach. Reliability and validity were evaluated by examining internal consistency via Cronbach’s alpha and composite reliability (CR), as well as convergent validity through standardized factor loadings and average variance extracted (AVE), in line with established guidelines [55–57]. Discriminant validity was assessed using the Fornell–Larcker criterion by comparing the square roots of AVE with inter-construct correlations [56] and was further verified using the heterotrait–monotrait ratio (HTMT) criterion.

Structural equation modeling (SEM) was employed to test the measurement model and examine the proposed relationships [58]. Model fit was evaluated using χ²/df, goodness-of-fit index (GFI), adjusted GFI (AGFI), normed fit index (NFI), comparative fit index (CFI), Tucker–Lewis index (TLI), and root mean square error of approximation (RMSEA), followed by an examination of path coefficients and their statistical significance [59]. The structural model was evaluated by examining standardized path coefficients, significance levels, explained variance (R²), and indirect effects. Given the departure from multivariate normality, bootstrapping with 5,000 resamples was used to obtain bias-corrected confidence intervals (CIs) for the structural path estimates and indirect effects.

## Results

### Descriptive statistics

The mean, standard deviation, skewness, and kurtosis were computed to examine the distributional properties of the variables. As shown in Table 3, the mean values ranged from 3.176 to 3.424, indicating moderate levels across all constructs. Among the constructs, GenAI Use Continuance Intention had the highest mean (M = 3.424), whereas GenAI Basic Understanding had the lowest mean (M = 3.176). Both values were above the midpoint of the measurement scale.

**Table 3:** Descriptive statistics.

Construct	*n*	Mean	Std. Deviation	Skewness	Kurtosis
**GenAI Basic Understanding**	615	3.176	1.064	−.269	−.620
**GenAI in Teaching**	615	3.311	1.020	−.424	−.322
**GenAI in Research**	615	3.233	1.018	−.344	−.319
**GenAI Professional Engagement**	615	3.245	1.012	−.310	−.374
**Perceived Usefulness**	615	3.218	1.090	−.366	−.631
**Perceived Ease of Use**	615	3.353	1.013	−.313	−.655
**GenAI Use Continuance Intention**	615	3.424	1.094	−.401	−.554

The standard deviations ranged from 1.012 to 1.094, indicating a moderate degree of variability in participants’ responses across the constructs. All skewness values were negative and relatively close to zero, ranging from −.424 to −.269, suggesting a slight concentration of responses toward higher scale values while maintaining relatively symmetric distributions. Kurtosis values ranged from −.655 to −.319, all falling within the commonly used ±2 criterion, indicating no substantial univariate departures from normality [60]. However, the assessment of multivariate normality indicated a departure from multivariate normality. Therefore, although the variables demonstrated acceptable univariate distributional properties, 5,000 bootstrap replications were employed in the SEM analysis to obtain robust estimates and confidence intervals.

### Common method bias

The first factor accounted for 28.386% of the total variance, which was below the recommended threshold of 50% [54]. This result suggests that no single factor dominated the variance explained by the measurement items, indicating that common method bias (CMB) was unlikely to pose a serious threat to the findings. Although Harman’s single-factor test has been widely used, it has limitations in detecting CMB.

To assess potential CMB further, a ULMC approach was taken by adding a common method factor to the measurement model. As shown in Table 4, the inclusion of the common method factor resulted in only marginal changes in model fit. Specifically, the changes in fit indices were small (ΔRMSEA = .002, ΔSRMR = .0011, ΔCFI = −.006, ΔGFI = −.011, ΔIFI = −.006, and ΔTLI = −.005). Overall, these small changes provide no strong evidence that common method bias substantially affected the measurement model or the observed relationships.

**Table 4:** Results of the ULMC test.

Model	χ²/df	RMSEA	SRMR	CFI	GFI	IFI	TLI
**Original measurement model**	1.768	.035	.0273	.957	.873	.957	.955
**ULMC model**	1.681	.033	.0262	.963	.884	.963	.960
**Difference (Δ)^a^**	—	.002	.0011	−.006	−.011	−.006	−.005

^a^Δ = Original measurement model − ULMC model.

### Reliability and validity analysis

All constructs demonstrated excellent internal consistency, with Cronbach’s alpha values ranging from.880 to.960, exceeding the recommended threshold of.70 [61]. Specifically, the reliability coefficients were.930 for GenAI-BU,.951 for GenAI-TE,.928 for GenAI-RE,.929 for GenAI-PE,.914 for PU,.880 for PEOU, and.960 for GenAI-UCI. These results indicate high internal consistency across all constructs.

As an initial assessment of factorability, the Kaiser–Meyer–Olkin value was.946, surpassing the recommended threshold of 0.80 and indicating excellent sampling adequacy [62]. Bartlett’s test of sphericity was statistically significant (χ² = 25,755.739, df = 1,485, *p*< .001), indicating that the correlation matrix was not an identity matrix and was suitable for factor analysis.

The measurement model demonstrated good fit. The chi-square to degrees of freedom ratio (χ²/df = 1.768) was below the recommended threshold of 3.0, indicating an acceptable fit [63]. The RMSEA was.035, well below the widely accepted threshold of.08, indicating good model fit. The incremental fit indices, including IFI (.957), TLI (.955), and CFI (.957), all exceeded the recommended threshold of.90, further supporting the adequacy of the measurement model [64].

Convergent validity was assessed using standardized factor loadings, average variance extracted (AVE), and composite reliability (CR). As shown in Table 5, all standardized factor loadings ranged from.647 to.885, exceeding the recommended threshold of.50, indicating adequate associations between the observed indicators and their intended latent constructs [55]. The AVE values ranged from.554 to.750, all exceeding the recommended threshold of.50, indicating that each construct accounted for more than half of the variance in its indicators [56]. The CR values ranged from.881 to.960, exceeding the recommended threshold of 0.80 and indicating satisfactory internal consistency [57]. Together, these results provide evidence of adequate convergent validity and reliability of the measurement model.

**Table 5:** Factor loadings and convergent validity.

Construct	Code	Factor Loading	Cronbach’s alpha	CR	AVE
**GenAI Basic Understanding**	GenAI-BU1	.812	.930	.930	.657
	GenAI-BU2	.815			
	GenAI-BU3	.847			
	GenAI-BU4	.789			
	GenAI-BU5	.787			
	GenAI-BU6	.795			
	GenAI-BU7	.826			
**GenAI in Teaching**	GenAI-TE1	.793	.951	.951	.618
	GenAI-TE2	.781			
	GenAI-TE3	.776			
	GenAI-TE4	.794			
	GenAI-TE5	.799			
	GenAI-TE6	.813			
	GenAI-TE7	.771			
	GenAI-TE8	.786			
	GenAI-TE9	.772			
	GenAI-TE10	.787			
	GenAI-TE11	.773			
	GenAI-TE12	.788			
**GenAI in Research**	GenAI-RE1	.789	.928	.929	.619
	GenAI-RE2	.799			
	GenAI-RE3	.759			
	GenAI-RE4	.802			
	GenAI-RE5	.797			
	GenAI-RE6	.749			
	GenAI-RE7	.818			
	GenAI-RE8	.781			
**GenAI Professional Engagement**	GenAI-PE1	.784	.929	.929	.622
	GenAI-PE2	.808			
	GenAI-PE3	.804			
	GenAI-PE4	.765			
	GenAI-PE5	.774			
	GenAI-PE6	.778			
	GenAI-PE7	.799			
	GenAI-PE8	.794			
**Perceived Usefulness**	PU1	.796	.914	.905	.613
	PU2	.768			
	PU3	.780			
	PU4	.786			
	PU5	.751			
	PU6	.815			
**Perceived Ease of Use**	PEOU1	.801	.880	.881	.554
	PEOU2	.738			
	PEOU3	.647			
	PEOU4	.757			
	PEOU5	.714			
	PEOU6	.798			
**GenAI Use Continuance Intention**	GenAI-UCI1	.884	.960	.960	.750
	GenAI-UCI2	.865			
	GenAI-UCI3	.885			
	GenAI-UCI4	.847			
	GenAI-UCI5	.861			
	GenAI-UCI6	.862			
	GenAI-UCI7	.868			
	GenAI-UCI8	.855			

Discriminant validity was evaluated using the Fornell–Larcker criterion by comparing the square roots of the AVE with inter-construct correlations. Table 6 presents the square roots of the AVE on the diagonal, ranging from.744 to.866. In each case, the square root of the AVE exceeded the correlations between the corresponding construct and all other constructs, supporting discriminant validity. To further assess discriminant validity, the HTMT criterion was examined. As shown in Table 7, all HTMT values were below the recommended threshold of 0.85, providing further evidence of adequate discriminant validity among the constructs.

**Table 6:** Correlation and the square root of AVE.

Construct	AVE	GenAI Basic Understanding	GenAI in Teaching	GenAI in Research	GenAI Professional Engagement	Perceived Usefulness	Perceived Ease of Use	GenAI Use Continuance Intention
**GenAI Basic Understanding**	.657	**.811**						
**GenAI in Teaching**	.618	.368***	**.786**					
**GenAI in Research**	.619	.479***	.477***	**.787**				
**GenAI Professional Engagement**	.622	.316***	.363***	.368***	**.789**			
**Perceived Usefulness**	.613	.387***	.433***	.413***	.350***	**.783**		
**Perceived Ease of Use**	.554	.083	−.067	.036	−.080	.132**	**.744**	
**GenAI Use Continuance Intention**	.750	.251***	.289***	.329***	.387***	.282***	.108*	**.866**

The square roots of AVE are presented on the diagonal (in bold), while the correlations between latent constructs are shown on the off-diagonal. ***p < 0.001, **p< 0.01, *p < 0.05.

**Table 7:** HTMT values for discriminant validity assessment.

	GenAI Use Continuance Intention	GenAI Professional Engagement	GenAI in Research	GenAI in Teaching	GenAI Basic Understanding	Perceived Ease of Use	Perceived Usefulness
**GenAI Use Continuance Intention**							
**GenAI Professional Engagement**	.387						
**GenAI in Research**	.330	.368					
**GenAI in Teaching**	.289	.363	.478				
**GenAI Basic Understanding**	.252	.314	.482	.369			
**Perceived Ease of Use**	.111	.079	.052	.069	.088		
**Perceived Usefulness**	.280	.353	.416	.438	.387	.138	

### Model fit and hypotheses tests

Structural equation modeling was conducted using AMOS 26.0 with the maximum likelihood (ML) estimation method. All measurement items were measured using a five-point Likert scale and were treated as continuous indicators in the SEM analysis. Prior to SEM analysis, the data were examined for missing values and distributional assumptions. No missing data were identified. However, the assessment of multivariate normality indicated a departure from multivariate normality. Therefore, 5,000 bootstrap replications were employed to obtain robust estimates and confidence intervals. The structural model demonstrated an overall acceptable fit. As shown in Table 8, the chi-square to degrees of freedom ratio (χ²/df = 2.118) was below the recommended threshold of 3.0. Both GFI (.846) and AGFI (.833) exceeded the criterion of.80. The NFI (.887) was slightly below the recommended criterion of.90. In contrast, TLI (.934) and CFI (.937) exceeded.90, while RMSEA (.043) was below.06. Taken together, the overall pattern of fit indices indicated an acceptable fit of the structural model to the data.

**Table 8:** Structural model fit indices.

Goodness-of-Fit Measure	Recommend Value	Result	Interpretation
**χ²/df**	< 3	2.118	good
**Goodness-of-fit Index**	≥ 0.80	.846	acceptable
**Adjusted Goodness-of-fit Index**	≥ 0.80	.833	acceptable
**Normalized Fit Index**	≥ 0.90	.887	slightly below criterion
**Tucker–Lewis Index**	≥ 0.90	.934	good
**Comparative Fit Index**	≥ 0.90	.937	good
**Root Mean Square Error of Approximation**	≤ 0.06	.043	good

**Fig 3:**
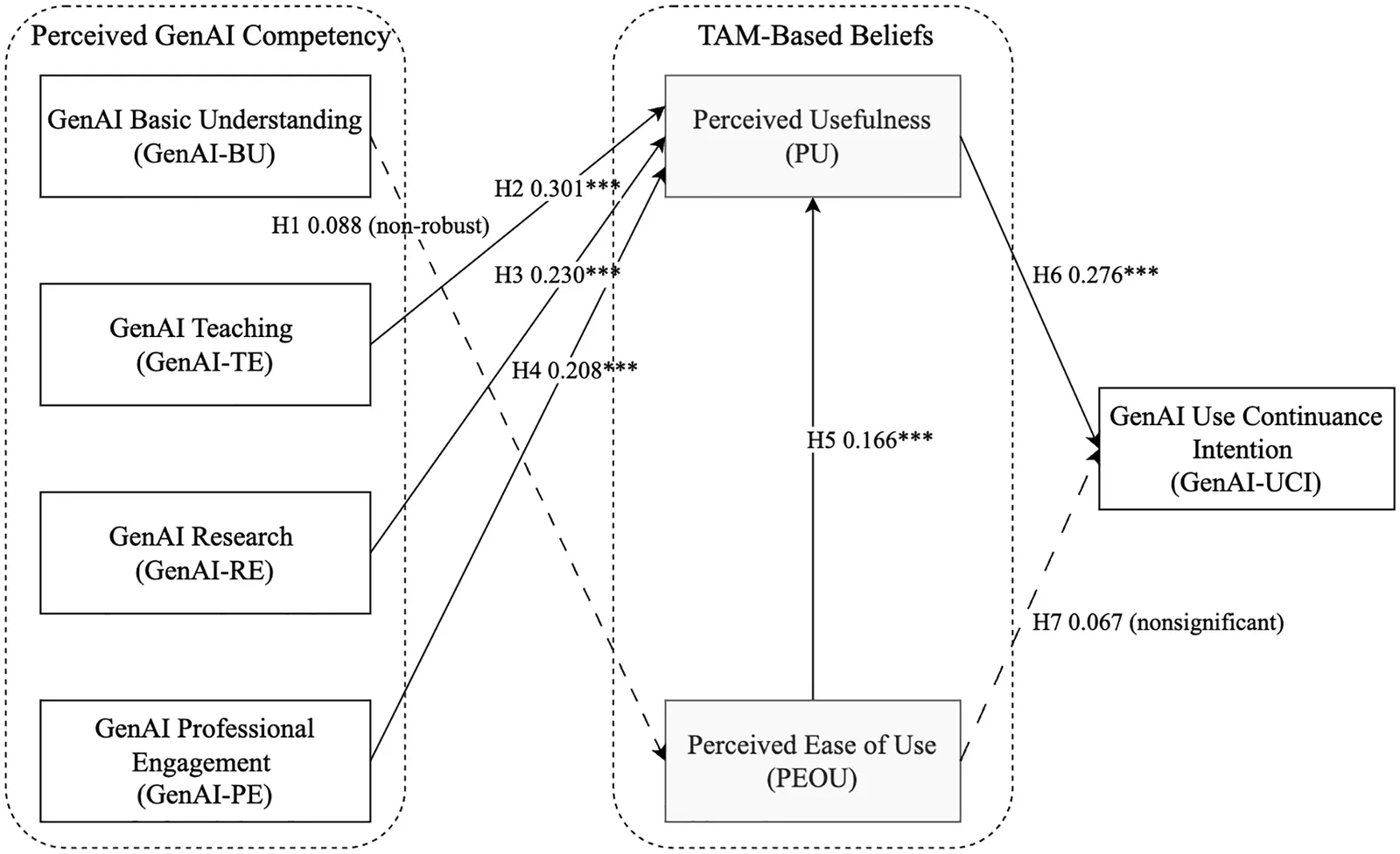
Structural model and hypothesis testing results. *** *p* < 0.001. “Non-robust” indicates that the 95% bias-corrected bootstrap confidence interval included zero despite p = .048 in the conventional significance test. “Nonsignificant” indicates *p* ≥ 0.05.

The path analysis investigated the interrelationships among the proposed constructs. Fig 3 depicts the structural model incorporating GenAI competencies (GenAI-BU, GenAI-TE, GenAI-RE, and GenAI-PE) alongside TAM-based beliefs (PEOU and PU) to predict GenAI-UCI. In total, six of the seven hypotheses were supported. The structural model explained 0.8% of the variance in PEOU, 21.5% in PU, and 8.7% in GenAI-UCI. Although the association between GenAI-BU and PEOU was statistically significant (β = .088, *p* = .048), its magnitude was small. The modest R² for GenAI-UCI also indicates that most of the variance in continuance intention remains unexplained by the current model. Thus, the findings provide evidence of specific associations within the proposed model rather than a comprehensive or principal mechanism underlying GenAI continuance intention.

The SEM results (Table 9) provided support for H2–H6, whereas H7 was not supported. For H1, the conventional significance test indicated a marginally significant association between GenAI-BU and PEOU (β = .088, p = .048); however, the 95% bias-corrected bootstrap confidence interval included zero [−.00003,.17922], indicating that this association was not robust across inferential procedures. Although the conventional significance test indicated a statistically significant association, the effect size was small (β = .088), and the p-value was close to the conventional.05 significance threshold. Therefore, the GenAI-BU → PEOU association should be interpreted cautiously and is considered not robustly supported. GenAI-TE, GenAI-RE, and GenAI-PE were positively associated with PU (β = .301,.230, and.208, respectively; all p < .001). PEOU was also positively associated with PU (β = .166, p < .001), and PU was positively associated with GenAI-UCI (β = .276, p < .001). In contrast, the direct association between PEOU and GenAI-UCI was not significant (β = .067, p = .114). These findings suggest that the different dimensions of perceived GenAI competence may be associated with different TAM beliefs within the hypothesized model: GenAI-BU showed a small and statistically non-robust association with PEOU, whereas GenAI-TE, GenAI-RE, and GenAI-PE were positively associated with PU. Furthermore, PEOU was positively associated with PU, whereas PU, but not PEOU, showed a significant direct association with GenAI-UCI.

**Table 9:** Structural model results and bootstrap confidence intervals.

Code	Path	Hypothesized Direction	*B* ^*a*^	SE^b^	CR^c^	*p*	β^d^	95% Bias-Corrected CI^e^	Decision
**H1**	GenAI-BU → PEOU	Positive	.087	.044	1.973	0.048	.088	[−.00003,.17922]	Not robustly supported
**H2**	GenAI-TE → PU	Positive	.302	.047	6.410	<.001	.301	[.1777,.4210]	Supported
**H3**	GenAI-RE → PU	Positive	.240	.049	4.906	<.001	.230	[.1050,.3546]	Supported
**H4**	GenAI-PE → PU	Positive	.214	.046	4.692	<.001	.208	[.0949,.3187]	Supported
**H5**	PEOU → PU	Positive	.161	.040	3.994	<.001	.166	[.0672,.2533]	Supported
**H6**	PU → GenAI-UCI	Positive	.298	.044	6.765	<.001	.276	[.1801,.3642]	Supported
**H7**	PEOU → GenAI-UCI	Positive	.070	.045	1.583	.114	.067	[−.0217,.1628]	Unsupported

^a^*B* = unstandardized estimate.

^b^SE = standard error.

^c^CR = critical ratio.

^d^β = standardized path coefficient.

^e^CI = bias-corrected bootstrap confidence interval based on 5,000 bootstrap resamples.

To examine the indirect associations between GenAI competencies and GenAI-UCI further, a bootstrapping analysis with 5,000 resamples was conducted. The results of the indirect effects analysis based on 95% bias-corrected CIs are presented in Table 10. The results indicated that GenAI-BU, GenAI-TE, GenAI-RE, and GenAI-PE all had significant indirect effects on GenAI-UCI through the proposed TAM-based pathways, as the corresponding 95% bias-corrected CIs did not include zero. Among these indirect effects, GenAI-TE showed the largest standardized indirect effect (β = .083), followed by GenAI-RE (β = .063), GenAI-PE (β = .057), and GenAI-BU (β = .010). Although the indirect association of GenAI-BU was statistically detectable, its magnitude was very small (β = .010), suggesting that its practical significance was limited.

**Table 10:** Results of bootstrapped indirect effects analysis.

Predictor	Outcome	Indirect Effect (β)	95% Bias-Corrected CI
**GenAI-BU**	GenAI-UCI	.010	[.0001,.0306]
**GenAI-TE**	GenAI-UCI	.083	[.0452,.1342]
**GenAI-RE**	GenAI-UCI	.063	[.0266,.1127]
**GenAI-PE**	GenAI-UCI	.057	[.0229,.1059]

### Sensitivity analysis

To assess whether the structural findings were sensitive to the potentially overlapping content between GenAI Professional Engagement and GenAI Use Continuance Intention, a sensitivity analysis was conducted by removing GenAI-UCI3 (“I plan to attend more workshops about Generative AI in education”), which showed the clearest conceptual overlap with the GenAI-PE item concerning participation in GenAI workshops. The revised model demonstrated comparable fit to the original model (χ²/df = 2.151, IFI = .935, TLI = .932, CFI = .935, RMSEA = .043). Importantly, all structural relationships retained the same direction and significance pattern as in the original model. GenAI-BU remained positively associated with PEOU (β = .087, *p* = .049), while GenAI-TE, GenAI-RE, and GenAI-PE remained positively associated with PU (β = .302,.240, and.214, respectively; all *p* < 0.001). PEOU remained positively associated with PU (β = .161, *p* < 0.001), and PU remained positively associated with GenAI-UCI (β = .297, *p* < 0.001), whereas the association between PEOU and GenAI-UCI remained nonsignificant (β = .066, *p* = .141). These results suggest that the substantive conclusions were robust to the removal of the potentially overlapping GenAI-UCI3 item.

## Discussion

This study combines GenAI-related competencies with TAM to examine university educators’ intention to continue using GenAI within the context of digital transformation in higher education [15]. The SEM analysis provides insights into the associations between different dimensions of perceived GenAI competency, teachers’ technology acceptance beliefs, and continuance intention. Unlike traditional TAM studies that primarily emphasize PU and PEOU as key antecedents of technology acceptance and usage intention [15,65], this study extends the TAM framework by examining how domain-specific perceived competencies are associated with teachers’ technology beliefs and continuance intention. The findings suggest that GenAI acceptance may be associated not only with technological perceptions but also with users’ perceived capability to integrate AI into professional practices [66].

The SEM results show that PU is a key cognitive factor associated with GenAI-UCI. Perceived competencies related to teaching, research, and professional engagement were positively associated with PU, indicating that teachers’ evaluations of GenAI may be particularly related to its relevance to academic and professional tasks. In contrast, PEOU did not show a direct association with continuance intention [67], although it remained positively related to PU [68]. This pattern suggests that ease of use may be related to teachers’ evaluations of GenAI, whereas continuance intention was more closely associated with perceived usefulness in the present model [69]. Although the association between GenAI-BU and PEOU was statistically significant in the conventional significance test, the 95% bias-corrected bootstrap confidence interval included zero, indicating that this association was not robust across inferential procedures. The effect was also small (β = .088), and should therefore be interpreted with caution. Similarly, the model explained a relatively modest proportion of variance in GenAI-UCI, indicating that substantial variation in continuance intention remains unexplained by the current model. These findings should therefore be interpreted as evidence of specific associations within the proposed TAM-based model rather than as evidence of a comprehensive pattern of associations underlying GenAI continuance intention. This pattern is broadly consistent with prior technology acceptance research suggesting that perceived usefulness may be particularly relevant to continued technology use, whereas perceived ease of use may play a more prominent role during earlier stages of technology engagement [70]. These findings suggest that, in this sample, intention to continue using GenAI may be more closely associated with perceived academic value than with ease of operation [71]. This interpretation is also broadly consistent with previous TAM studies indicating that PU may be a stronger predictor of continuance intention than PEOU once users have gained familiarity with a technology [67,68]. It also aligns with recent studies on GenAI adoption, which suggest that teachers’ intention to continue using AI tools may be related to perceived instructional and professional benefits [72]. Furthermore, the bootstrapped indirect effects analysis showed significant indirect associations of all four GenAI competency dimensions with GenAI-UCI through the proposed TAM-based pathways. However, the indirect association involving GenAI-BU was very small (β = .010), indicating limited practical significance. Thus, its statistical detectability should not be interpreted as evidence of a substantively meaningful or strong mechanism linking GenAI-BU to GenAI-UCI.

The findings suggest different patterns of association between competency dimensions and TAM beliefs within the hypothesized structural model. Perceived GenAI basic understanding showed a small and non-robust association with PEOU, whereas perceived GenAI teaching, research, and professional engagement were positively associated with PU. These findings support the specified associations between particular competency dimensions and TAM beliefs, but they do not establish that these dimensions operate through empirically distinct psychological pathways. Rather, they suggest that different aspects of perceived GenAI competency may be relevant to different technology acceptance beliefs. The significant indirect associations of all four competency dimensions with GenAI-UCI through the proposed TAM-based pathways further suggest that these competencies may be relevant to continuance intention within the specified model. Because alternative cross-paths were not estimated, these findings should be interpreted as support for the hypothesized associations rather than as evidence that the competency dimensions are empirically distinct in their relationships with TAM beliefs. From a theoretical perspective, these findings highlight the potential relevance of domain-specific perceived competencies for understanding teachers’ technology acceptance beliefs. Rather than treating perceived GenAI competency as a homogeneous capability, the findings provide preliminary evidence that its different dimensions may be associated with different aspects of technology acceptance. Future research could examine whether these associations remain when competing relationships among competency dimensions and TAM beliefs are explicitly modeled, as well as whether the pattern varies across disciplines, institutional contexts, or stages of GenAI use.

The Chinese higher education context provides additional insight into these patterns. Institutional environments often emphasize research productivity, performance outcomes, and collective norms [68], which may help explain why teachers’ perceived competencies related to teaching, research, and professional engagement were associated with PU in the present sample. These institutional characteristics may provide a possible contextual explanation for why teachers may place greater emphasis on the practical value of GenAI for teaching and research activities. This contextual interpretation is particularly relevant in Chinese higher education, where faculty evaluation systems often place substantial emphasis on research performance and teaching quality [73]. Consequently, teachers may perceive GenAI as valuable when it supports measurable professional outcomes rather than simply improving technological convenience. At the same time, the nonsignificant direct association between PEOU and GenAI-UCI suggests that ease of use alone may not be sufficient to explain continuance intention in this sample, although PEOU remained positively associated with PU. Institutional policies and organizational expectations may also be related to teachers’ evaluations of GenAI beyond the individual-level factors examined in this study. Future comparative studies across different national and institutional contexts could further clarify how contextual factors are associated with technology acceptance and continuance intention.

Taken together, these findings indicate that teachers’ continuance intention toward GenAI is associated with TAM-based beliefs, particularly PU, while the explanatory power of the current model remains modest. From a practical perspective, higher education institutions may consider professional development initiatives that move beyond technical training and provide opportunities for meaningful engagement with GenAI in teaching, research, and professional activities. As a tentative implication, professional development could be tailored to teachers’ differing levels of GenAI experience. For example, foundational training may be relevant for teachers with limited GenAI experience, whereas pedagogical integration, research applications, and professional engagement may be relevant areas for more experienced users. However, these differentiated recommendations were not directly tested in the present study and should therefore be regarded as practical suggestions rather than empirical findings. Future research could examine whether professional development needs differ across levels of GenAI experience and whether such differences are associated with continuance intention.

## Limitations and future research

Numerous limitations must be acknowledged. The study’s cross-sectional design limits the ability to examine changes in continuance intention over time. Furthermore, the study is limited to Chinese higher education institutions, which may restrict the generalizability of the findings to other cultural and institutional contexts. Moreover, because participants were recruited through convenience sampling and were required to have prior familiarity with AI tools, the sample primarily represents university teachers who already have some exposure to GenAI. Therefore, the findings may not be directly generalized to university teachers with limited or no experience with GenAI. Additionally, the structural model demonstrated modest explanatory power for GenAI continuance intention, suggesting that other individual, technological, and contextual factors may also be associated with teachers’ intention to continue using GenAI. Although the sensitivity analysis suggested that the findings were robust after removing the most conceptually overlapping GenAI-UCI item, some conceptual proximity between professional engagement and continuance intention may remain because both constructs involve future-oriented GenAI-related activities.

Future research might adopt longitudinal designs to investigate how teachers’ GenAI competencies, perceptions, and continuance intentions develop over time. Comparative analyses across multiple cultural and institutional contexts would enhance understanding of contextual factors associated with GenAI use and continuance intention. Qualitative approaches could further uncover how teachers integrate GenAI into daily academic practices and how contextual conditions are related to their experiences. Future studies could also incorporate additional predictors, such as institutional support, social influence, trust, AI-related anxiety, and prior experience, to develop a more comprehensive understanding of GenAI continuance intention. Moreover, future research could examine whether the associations of different competency dimensions with continuance intention vary across disciplines, career stages, or institutional contexts.

## Conclusions

This research enhances understanding of how perceived GenAI competency is associated with technology acceptance beliefs (PU and PEOU), and how these beliefs are further associated with university teachers’ intention to continue using GenAI in higher education. The findings contribute to existing knowledge by examining how different dimensions of perceived GenAI competency are associated with teachers’ continuance intention through technology acceptance beliefs within the proposed model.

The study makes three primary contributions. First, it extends the TAM framework by incorporating perceived GenAI competency as an antecedent of technology acceptance beliefs. The findings indicate that different dimensions of perceived competency are associated with different TAM beliefs within the hypothesized model: foundational GenAI understanding showed a small and non-robust association with PEOU, whereas teaching, research, and professional engagement competencies were positively associated with PU. Second, it contributes to understanding GenAI continuance intention by examining the TAM-based indirect associations between perceived competencies and continuance intention. The findings indicate that the four competency dimensions were indirectly associated with continuance intention through the proposed TAM-based pathways. Third, it contributes to educational technology adoption research by suggesting that teachers’ perceived competencies in teaching, research, and professional engagement may be relevant to GenAI continuance intention through their association with perceived usefulness.

In terms of practical implications, the findings suggest that intention to continue using GenAI may be related to its integration into teaching and research activities. Instead of focusing solely on usability or technical training, higher education institutions could prioritize meaningful academic engagement with GenAI. Professional development may consider areas such as foundational GenAI knowledge, pedagogical integration, research applications, and responsible AI practices, although these areas were not directly compared or tested in the present study. These suggestions should therefore be regarded as tentative implications rather than empirically demonstrated differences in professional development needs across teachers.

Overall, this study suggests that university teachers’ continuance intention toward GenAI is associated with their perceptions of its usefulness within academic practices. However, the modest explanatory power of the proposed model indicates that continuance intention may also be associated with additional factors beyond those examined in this study. The findings should therefore be interpreted as evidence of specific associations within the proposed model rather than as establishing a comprehensive explanation of GenAI continuance intention.
